# Online Handwritten Signature Verification Method Based on Uni-Feature Correlation Coefficient between Signatures

**DOI:** 10.3390/s23239341

**Published:** 2023-11-22

**Authors:** Ruonan Liu, Yizhong Xin

**Affiliations:** School of Information Science and Engineering, Shenyang University of Technology, Shenyang 110870, China; lrn_123@smail.sut.edu.cn

**Keywords:** online handwritten signature verification, uni-feature, correlation coefficient, multi-feature fusion

## Abstract

Online handwritten signature verification is a crucial direction of research in the field of biometric recognition. Recently, many studies concerning online signature verification have attempted to improve performance using multi-feature fusion. However, few studies have provided the rationale for selecting a certain uni-feature to be fused, and few studies have investigated the contributions of a certain uni-feature in the multi-feature fusion process. This lack of research makes it challenging for future researchers in related fields to gain inspiration. Therefore, we use the uni-feature as the research object. In this paper, the uni-feature is one of the *X* and *Y* coordinates of the signature trajectory point, pen pressure, pen tilt, and pen azimuth feature. Aiming to solve the unequal length of feature vectors and the low accuracy of signature verification when using uni-features, we innovatively introduced the idea of correlation analysis and proposed a dynamic signature verification method based on the correlation coefficient of uni-features. Firstly, an alignment method of two feature vector lengths was proposed. Secondly, the correlation coefficient calculation formula was determined by analyzing the distribution type of the feature data, and then the correlation coefficient of the same uni-feature between the genuine signatures or between the genuine and forged signatures was calculated. Finally, the signature was verified by introducing a Gaussian density function model and combining it with the signature verification discrimination threshold. Experimental results showed that the proposed method could improve the performance of dynamic signature verification based on uni-features. In addition, the pen pressure feature had the best signature verification performance, with the highest signature verification accuracy of 93.46% on the SVC 2004 dataset.

## 1. Introduction

Signature verification can be divided into offline and online signature verification [1]. Offline signature verification often determines whether a signature belongs to the same writer by analyzing static features such as font structure or stroke order in the signature image. In contrast, online signature verification generally verifies performance by extracting dynamic features such as pen pressure, pen tilt, or speed in the signature process. Yang et al. [2] discovered that replicating dynamic features is more challenging than replicating static features. Furthermore, they also found that the performance of online signature verification is generally better than that of offline signature verification [3]. In recent years, researchers have proposed various methods to enhance the performance of online signature verification. For example, Chandra et al. [4] proposed a local weighted classification method based on dynamic features. The results showed that the false acceptance rate (*FAR*) and false rejection rate (*FRR*) were 11.8% and 2%, respectively. Considering the potential impact of limited training samples on signature verification, Vorugunti et al. [5] proposed a dynamic signature verification framework using depth-separable convolution. This approach facilitates dimensionality reduction in feature data, leading to enhanced accuracy in signature verification. Shen et al. [6] developed a Siamese network framework integrated with a multi-scale attention mechanism to extract efficient features from a limited number of signature samples. The findings demonstrated that the equal error rate (*EER*) for signature verification in the MCYT-100 dataset was 6.57%. Ahrabian et al. [7] integrated an automatic encoder with a Siamese neural network to establish a global feature framework. The results showed that this method had better performance on large datasets. Xia et al. [8] proposed a feature selection method that combined the full factorial design method and the optimal orthogonal design method. The results showed that the equal error rate of signature verification on the MCYT-100 dataset was 2.17%. Sharma et al. [9] combined the cost matrix with the dynamic time warping algorithm and introduced a spacing parameter for feature extraction to improve the accuracy of signature verification. Okawa et al. [10] proposed a dynamic time warping algorithm based on local and global weighting. The algorithm first acquires the average template set of reference samples using a dynamic time warping algorithm based on the Euclidean center of gravity. Then, it computes the distances between templates and test samples using local and global weighting, followed by signature verification. He et al. [11] extracted the curvature and torsion features and calculated the distance between the test signature and the template using Hausdorff distance. Bhowal et al. [12] designed an integrated system that combined seven feature classifiers. The results showed a signature verification accuracy of 98.43% and 97.87% when tested on the SVC2004 and MCYT-100 datasets, respectively.

Although there are many online handwritten signature verification methods, a further review of the literature reveals that existing online handwritten signature verification methods generally rely on multi-feature fusion. For example, Jiang et al. [13] fused 12 dynamic features, including pen pressure, pen tilt, and acceleration, and improved the traditional dynamic time warping method to enhance signature verification performance. Anikin et al. [14] fused five features, including the *X* and *Y* coordinates of the signature trajectory point, pen pressure, pen tilt, and pen azimuth. Additionally, they also developed a user authentication framework for online handwritten signature verification. Wu et al. [15] also fused the above five dynamic features. However, they used the Siamese network to improve the accuracy of signature verification. Saleem et al. [16] fused the *X* and *Y* coordinates of the signature trajectory point and pen pressure feature and combined the dynamic time warping algorithm with the KNN algorithm to achieve signature verification. Foroozandeh et al. [17] also fused the above three features in their study and used the KNN algorithm and the SVM algorithm for signature verification. Abualghanam et al. [18] fused 134 features and fed them into a convolutional neural network. Parziale et al. [19] investigated signature verification performance with 15 different feature fusion methods. The results showed that the optimal performance for signature verification was achieved by fusing velocity, acceleration, and pen pressure features. Okawa et al. [20] fused seven features and proposed a local stability-weighted dynamic time warping algorithm for signature verification.

Previous researchers have focused on fusing multi-features. However, they have not examined the performance of signature verification with a certain uni-feature or explained the rationale behind feature fusion. This lack of research makes it challenging for future researchers in related fields to gain inspiration. Based on this, it is necessary to examine signature verification performance with a uni-feature. By investigating signature verification performance with a certain uni-feature, we can determine the importance of each feature in the signature verification process. If signature verification performs better with a certain uni-feature, it can be considered to increase the weight of the feature during multi-feature fusion. At present, few studies have investigated the performance of online signature verification with a uni-feature. In the literature, Lei et al. [21] investigated the performance of online signature verification using pen pressure and pen tilt features with a dynamic time warping algorithm. The results showed that the equal error rate of each feature on the SVC 2004 dataset was generally higher than 20%. Adamski et al. [22] conducted a study on the accuracy of signature verification using pen pressure, pen tilt, and pen azimuth features, respectively. The results showed the highest signature verification accuracy with the pen pressure feature. However, although a few studies have examined a certain uni-feature, the variety of features investigated is limited and signature verification performance is also poor. Therefore, to improve the performance of online signature verification with a certain uni-feature as much as possible and provide a reference for research related to signature verification with multi-feature fusion, we investigated five uni-features and introduced correlation analysis methods. In this paper, the uni-features used are as follows: *X* coordinate of the signature trajectory point, *Y* coordinate of the signature trajectory point, pen pressure, pen tilt, and pen azimuth. We implemented signature verification by calculating the correlation coefficient of the same uni-feature between the genuine signatures or between the genuine and forged signatures, respectively.

Currently, correlation analysis methods are widely used across various fields. For example, Garcia et al. [23] proposed a new automatic encoder classification method to recognize and classify human activities. They comparatively analyzed the effect of various features on human activity recognition performance, including the Pearson correlation coefficient, standard deviation, and mean value. Sverko et al. [24] utilized EEG to evaluate the inter-neuronal connectivity and measured the relationship between phase-locked values and weighted phase lag indices using the Pearson correlation coefficient. To aid biologists in expanding their understanding of the intricate processes behind cancer, Tanvir et al. [25] used the Pearson correlation coefficient to identify highly correlated gene pairs and constructed a gene co-expression network. Liu et al. [26] performed human posture correction by comparing the Pearson correlation coefficient between standard and measured postures. Bommisetty et al. [27] proposed a keyframe extraction method that combined the Pearson correlation coefficient and color moments. Mohapatra et al. [28] proposed a modified Pearson correlation coefficient to improve the limitations of the Pearson correlation coefficient in identifying two-dimensional fluorescent images of spherical cells. Nasir et al. [29] used the Pearson correlation coefficient to optimize redundant features and implemented document classification through deep neural networks. Friedman et al. [30] used the intra-class correlation coefficient to determine more stable biometrics. They found that the equal error rate after using the intra-class correlation coefficient was 2.1%. Luo et al. [31] built a brain function network based on a Pearson correlation coefficient matrix to improve the accuracy of feature classification with the brain–computer interface. Their findings indicated that classification accuracy could reach 88.67%. Zhang et al. [32] used the Pearson correlation coefficient and a random forest regression model to investigate the correlation between coronavirus disease and weather factors. The results showed that the Pearson correlation coefficient between daily confirmed cases and temperature was weak. Liu et al. [33] proposed a feature selection method based on an improved genetic algorithm that used the Pearson correlation coefficient to measure the contribution of each feature and thus determine the optimal feature. Esmailoghli et al. [34] extracted effective features by calculating Spearman’s correlation coefficient between nonlinear data. Sun et al. [35] found that Spearman’s correlation analysis could not be used for gene content prediction. Xue et al. [36] used Spearman’s correlation analysis to investigate the correlation between lung ultrasonography score and disease severity in patients with coronavirus pneumonia. Chamoun et al. [37] examined Spearman’s correlation coefficient between taste sensitivity and preference. The results showed that a greater sensitivity to certain flavors was associated with a reduced preference for those same flavors.

From the above literature, many studies have introduced Pearson correlation analysis or Spearman’s correlation analysis to improve the performance of recognition or classification. However, concerning online signature verification, few studies have introduced correlation analysis, and few studies have used the correlation coefficient of uni-features for signature verification. In the retrieved literature, Santos et al. [38] calculated the Pearson correlation coefficient between the *X* and *Y* coordinates of the signature trajectory point. Subsequently, they combined classification methods such as decision trees and support vector machines to realize online signature verification. Li et al. [39] identified genuine and forged signatures by calculating the Pearson correlation coefficient between the characteristics of pen pressure. However, they did not provide the false recognition rate, false rejection rate, or other indicators that can be used to evaluate the algorithm’s performance. Liu et al. [40] used the Pearson correlation coefficient for online signature verification. However, it is noteworthy that this method exclusively analyzed the performance of multi-feature fusion for signature verification and did not evaluate the efficiency of online signature verification using uni-features. Although these researchers performed signature verification using correlation analysis, they did not detail the exact process of using correlation analysis, nor did they examine the difference using various uni-features.

In summary, to enhance the performance of online signature verification using uni-features, investigate performance differences in signature verification with different uni-features, provide a contribution for each feature in signature verification, and provide a reference on multi-feature fusion, we innovatively introduce the idea of correlation analysis. First, the alignment method of two feature vector lengths is proposed. Then, the correlation analysis method is determined by judging the feature data distribution type, and the correlation coefficient of the same uni-feature between the genuine signatures or between the genuine and forged signatures is calculated. Finally, signature verification is carried out using the Gaussian density function model and combining it with the discriminant threshold.

## 2. Online Signature Verification Method Based on Uni-Features

**Figure 1:**
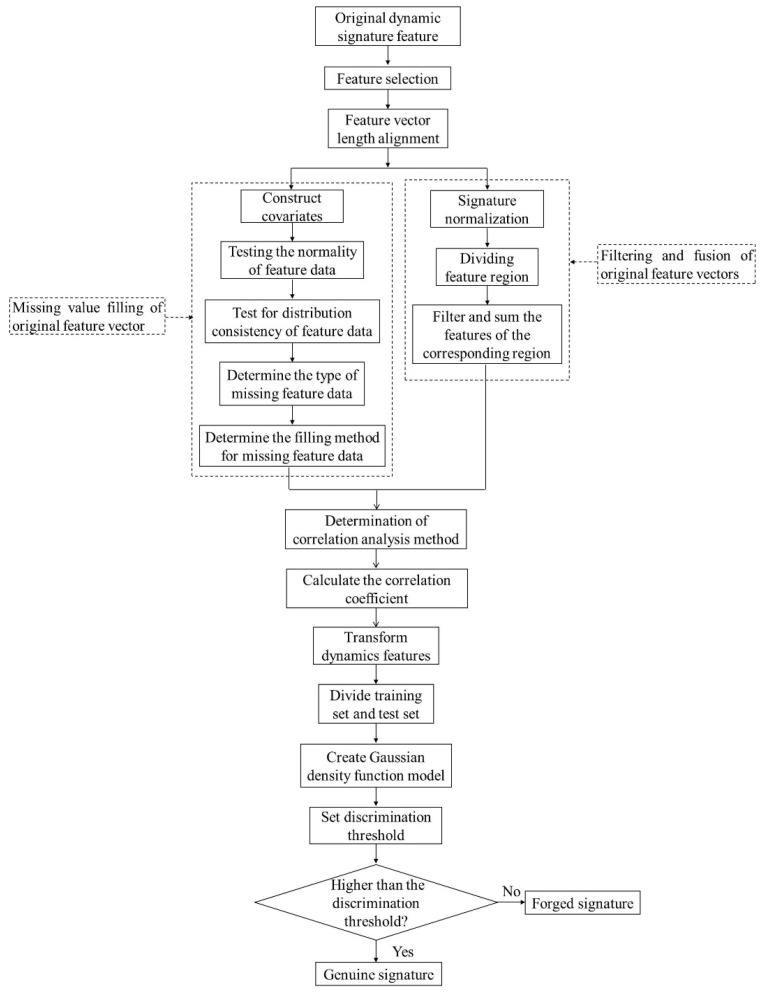
Technical route of online signature verification.

The technical road of the proposed method is shown in Figure 1. Firstly, the collected original dynamic signature features are selected to determine the uni-feature that needs to be subsequently processed. Then, the original feature vector length is aligned using the proposed “filling the missing value of the original feature vector” method and “filtering and fusion of the original feature vector” method. Subsequently, the correlation analysis method is determined using the normal distribution test method. The correlation coefficient of the same uni-feature between the genuine signatures or between the genuine and forged signatures is calculated. Secondly, the calculated correlation coefficient is used as a new dynamic feature. On this basis, the training set and the test set are divided, and then the function value is calculated combined with the Gaussian density function model. Finally, we set a threshold to distinguish between genuine and forged signatures.

### 2.1. Feature Selection

Taking into account signature content, writing conditions, data complementarity, and other relevant factors, we selected the xLongSignDB and SVC 2004 datasets, which represent the complexity of the real world as closely as possible. The xLongSignDB dataset [41,42] comprises signatures written by 29 writers over 15 months, including every writer’s 46 genuine signatures and 10 skilled forged signatures. The SVC 2004 dataset [43] consists of three subsets: *Sample*, *Task1*, and *Task2*. The *Task1* subset was not chosen for use in this paper because it only includes coordinate and time information. The *Sample* and *Task2* subsets, containing 45 writers’ 20 genuine signatures and 20 skilled forged signatures, were used in the paper.

The *Sample* subset and *Task2* subset of the SVC 2004 dataset and the xLongSignDB dataset provide the *X* and *Y* coordinates of the signature trajectory point, time, the current state of the pen (down or up), pen pressure (pressure exerted by the writer on the pen tip), pen tilt (angle between the pen body and the digitizing tablet), and pen azimuth (angle between the vertical projection of the pen body on the digitizing tablets and the north direction of the digitizing tablets) information for each sampling point of the signature. In order to improve the performance of dynamic signature verification with a uni-feature, we also referenced the feature selection strategy from some of the literature. Lei et al. [21] believe that a feature’s stability might affect signature verification performance. Consequently, they developed a feature stability model utilizing the feature distance’s mean and standard deviation. They investigated the stability of various features, including the *X* and *Y* coordinates of the signature trajectory point, pen pressure, pen tilt, and pen azimuth. Their findings revealed that the stability of the *X* and *Y* coordinate features was superior. Therefore, they concluded that *X* and *Y* coordinate features are more suitable for dynamic signature verification. In addition to the *X* and *Y* coordinate features, Lei et al. [21] also found that pen pressure, pen tilt, and pen azimuth were relatively unstable. Taking the pen pressure feature as an example, Lei et al. [21] believed that the pen pressure of a person might change significantly, making it difficult to determine the signature’s authenticity. However, they also found that when the changing trend of pen pressure of a signature was very similar to that of a genuine signature, the signature was more likely to be genuine. Moreover, Li et al. [39] also found that the distribution of pen pressure characteristics of genuine and forged signatures was significantly different, which indicated that these features might be used to distinguish between genuine and forged signatures.

Considering the above factors, we mainly examined online signature verification performance with the *X* coordinate (*X*), *Y* coordinate (*Y*), pen pressure (*P*), pen tilt (*T*), and pen azimuth features (*A*). To facilitate the subsequent representation, taking the pen pressure feature as an example, let *P* be the set of original pen pressure feature data of *m* signatures, that is, $P=\left\{p_{1},\;p_{2},\;p_{3},\ldots ,\;p_{m}\right\}$. $p_{m}$ is the original pen pressure feature vector of the *m*-th signature, and each original pen pressure feature vector can be expressed as $p_{m}={\left[p_{m1},\;p_{m2},\;p_{m3},\ldots ,\;p_{mn}\right]}^{⊤}$, in which $p_{mn}$ is the *n*-th original pen pressure feature data of the original pen pressure feature vector of the *m*-th signature.

### 2.2. Feature Vector Length Alignment Method

To realize dynamic signature verification according to a uni-feature, we propose a correlation analysis method for signature verification by calculating the correlation coefficients of a certain uni-feature between the genuine signatures or between the genuine and forged signatures, respectively. To calculate the correlation coefficient, all feature vectors must be processed to be of equal length. However, due to the significant level of unpredictability and personal interpretation involved in the signature process, signatures can be different each time, even when written by the same person. As a result, the length of the sample point sequence used to represent a signature may also differ, leading to unequal feature vector lengths. Thus, we propose two feature vector length alignment methods.

#### 2.2.1. Feature Vector Length Alignment Method by Filling the Missing Value according to Original Feature Vector Missing Situation

Taking the original pen pressure feature vectors $p_{1}$ and $p_{2}$ of the two genuine signatures of USER1 in the SVC 2004 dataset as an example, if the lengths of $p_{1}$ and $p_{2}$ are c and w, respectively, where c > w, then $p_{1}={\left[p_{11},\;p_{12},\ldots ,\;p_{1w},\;p_{1w+1},\ldots ,\;p_{1c}\right]}^{⊤}$ and $p_{2}={\left[p_{21},\;p_{22},\ldots ,\;p_{2w}\right]}^{⊤}$. Two strategies can be used to align the feature vector length without destroying the continuity of the original feature data. The first strategy is to delete the (${\left[p_{1w+1},\ldots ,\;p_{1c}\right]}^{⊤}$) part of $p_{1}$ directly. However, if the length of $p_{2}$ is less than the length of $p_{1}$ by more than 5%, the deletion may lead to a significant error result [44]. The second strategy is to fill the missing value (${\left[p_{2w+1},\;p_{2w+2},\ldots ,\;p_{2c}\right]}^{⊤}$) in $p_{2}$ so that $p_{2}={\left[p_{21},\;p_{22},\ldots ,\;p_{2w},\;p_{2w+1},\;p_{2w+2},\ldots ,\;p_{2c}\right]}^{⊤}$. There are multiple causes for missing data. Deng et al. [45] believed that missing experimental data are usually related to missing randomness in the user’s operation or systematic missing data during data collection. In this paper, missing data mainly result from the randomness and uncertainty surrounding the signature process. These factors resulted in data partially missing the original sampling point used to represent the signature in the signature datasets. Deng et al. [45] demonstrated that the filling method can be determined according to the type of missing data.

Generally, missing data types include *missing completely at random*, *missing at random*, and *missing not at random* [46]. When judging the missing data type, Sun et al. [47] identified that the assumption was gradually decreasing for *missing completely at random*, *missing at random*, and *missing not at random*, so the type of missing data could be tested from strong to weak. Sun et al. [47] also found that the type of missing data in the target variable can be indirectly determined by constructing covariates and examining whether the distributions of the components in the covariates are the same. If the distributions of the components in the covariates are the same, the missing data in the target variable are judged to be *missing completely at random*; if the distributions of the components in the covariates are not the same, it is necessary to build a regression model further to determine whether the missing data belongs to *missing at random*; and if the missing data is neither *missing completely at random* nor *missing at random*, the missing data is considered to be *missing not at random*. We will consider utilizing this method, taking $p_{1}$ and $p_{2}$ as examples, to first determine whether the missing data in the target variable $p_{2}$ are *missing completely at random*. Since $p_{1}$ and $p_{2}$ are feature vector pairs in the subsequent calculation of correlation coefficients, $p_{1}$ can be taken as a covariate and divided into two parts, (${\left[p_{11},\;p_{12},\ldots ,\;p_{1w}\right]}^{⊤}$) and (${\left[p_{1w+1},\ldots ,\;p_{1c}\right]}^{⊤}$), and then, whether the distribution of (${\left[p_{11},\;p_{12},\ldots ,\;p_{1w},\;p_{1w+1},\ldots ,\;p_{1c}\right]}^{⊤}$), (${\left[p_{11},\;p_{12},\ldots ,\;p_{1w}\right]}^{⊤}$), and (${\left[p_{1w+1},\ldots ,\;p_{1c}\right]}^{⊤}$) is the same can be investigated.

Before analyzing the consistency of the aforementioned sample distribution, it is crucial to assess whether the feature data conform to the normal distribution, as it is the fundamental assumption for many statistical methods [48,49,50]. In addition, the normal distribution test is different for different sample sizes. For sample sizes below 50, the normal distribution test employs the Shapiro–Wilk test (S-W). In contrast, if the sample size is 50 or more, the Kolmogorov–Smirnov test (K-S) is used for the same purpose [48,49]. Both methods mentioned above judge whether the current data distribution type meets the normal distribution, depending on whether the normal test result is greater than 0.05 [48,49]. If the feature data conform to the normal distribution, the *F*-test [50] is used to test the consistency of the feature vector distribution. Conversely, if the feature data do not conform to the normal distribution, the Kruskal–Wallis sample distribution consistency test (K-W) [50] is used to examine the consistency of the feature vector distribution.

In summary, to judge whether the missing data in the target variable $p_{2}$ are *missing completely at random*, it is necessary to examine whether the data distribution of the covariate $p_{1}$ (${\left[p_{11},\;p_{12},\ldots ,\;p_{1w},\;p_{1w+1},\ldots ,\;p_{1c}\right]}^{⊤}$) of the target variable $p_{2}$, the data part (${\left[p_{11},\;p_{12},\ldots ,\;p_{1w}\right]}^{⊤}$) corresponding to $p_{2}$ in the covariant $p_{1}$, and the data part (${\left[p_{1w+1},\ldots ,\;p_{1c}\right]}^{⊤}$ ) not corresponding to $p_{2}$ in the covariant $p_{1}$ are consistent. Suppose the distribution consistency test result is less than 0.05. In that case, this indicates that the three distributions are inconsistent [47]. The missing type of data in the target variable $p_{2}$ does not belong to the *missing completely at random*, and it is necessary to establish further a regression model to determine the type of the current missing data; otherwise, this indicates that their distributions are consistent, and the missing type of data in the target variable $p_{2}$ belongs to the *missing completely at random* type [47].

Based on the above testing steps, to determine the type of missing data for all original feature vectors in the dataset, we first conducted a normal distribution test for all feature data using the corresponding normal distribution test method according to the actual sample size. The results showed that all the original feature data did not conform to a normal distribution. Therefore, we conducted the K-W sample distribution consistency test on a total of 373,520 pairs of original feature vectors (46 genuine signatures × 46 genuine signatures × 5 features × 29 writers + 46 genuine signatures × 10 forged signatures × 5 features × 29 writers) in the xLongSignDB dataset and a total of 180,000 pairs of original feature vectors (20 genuine signatures × 20 genuine signatures × 5 features × 45 writers + 20 genuine signatures × 20 forged signatures × 5 features × 45 writers) in the SVC 2004 dataset. The K-W test results showed that the consistency test result of the sample distribution of a total of 452,816 pairs of original feature vectors in the two datasets was not less than 0.05. These results indicate that there was generally no significant difference between the distributions of these feature vectors and that these feature vectors of the missing data belonged to the *missing completely at random* type [47]. In addition, the consistency test results of 100,704 samples of the original feature vectors were less than 0.05, indicating significant differences between the distributions of a small number of feature vectors; that is, the distribution of feature vectors was inconsistent. For these feature vectors, the missing data type was determined by establishing a regression model between missing variables and covariates [47], as in Equation (1):

(1) $$\log it(\eta )=\log\frac{\eta }{1-\eta }=a+bx$$

where η is the missing probability and *x* is a covariate. If coefficient *b* in the model is not 0, the missing data type is *missing at random*. Otherwise, it is *missing completely at random* [47]. Based on this, after building regression models for the above 100,704 pairs of original feature vectors, the results showed that coefficient *b* in the model was not zero, so its missing type was *missing at random*.

When missing data are *missing completely at random* or *missing at random*, mean imputation (MEI) or multiple imputation (MI) can be selected to fill the data [45]. Among them, mean imputation needs to calculate the mean of the observed data in the feature vector containing missing values and use the mean as the imputation value to fill the missing part of the feature vector. On the other hand, multiple imputation needs to use the imputation model and the observed data to estimate multiple groups of values and then select the most appropriate group of imputation values through comprehensive analysis. Considering the efficiency issue, we chose the multiple imputation method based on chained equations that can save imputation time [51]. In addition, considering that the number of imputations may affect the performance of subsequent signature verification, pre-experiments were carried out to investigate the signature verification performance of three, five, and six imputations before the formal imputation in this paper. The results showed that when three imputations were carried out, the imputation speed was faster, but the accuracy was too low; when imputation was carried out five times, the accuracy was significantly improved. However, when imputation was performed six times, the accuracy was slightly improved, but there was no significant difference compared to that with five times, and the speed performance was significantly reduced. Based on the above results, we selected five imputations. Then, the average value of the five imputations was filled as the final content into the shorter feature vector with missing values after comparing the lengths of two feature vectors, so that its length was consistent with the length of another feature vector to realize the length alignment of two feature vectors. Furthermore, in order to compare the impact of different filling methods on the performance of signature verification, in addition to the above methods, we also investigated the method of filling the missing parts of the feature vector directly with zero.

Considering that the correlation analysis method varies with the distribution of the data to be analyzed, the distribution of the feature data after length alignment was investigated before calculating the correlation coefficient between the feature vectors. Pearson correlation analysis can be used if the feature data meet both the normal distribution and linear relationships; otherwise, Spearman’s correlation analysis is used [52,53]. We used the Kolmogorov–Smirnov (K-S) normal test on the feature vectors after length alignment and found that the normal test result was generally less than or equal to 0.05, indicating that the feature data after length alignment generally did not conform to the normal distribution. Therefore, we used Spearman’s correlation analysis to address this situation and conduct the subsequent analysis. Unlike Pearson correlation analysis, Spearman’s correlation analysis is a non-parametric statistical method that does not require samples to conform to a normal distribution and has a broader range of applications [52,53]. During the calculation of Spearman’s correlation coefficient, it is necessary to sort the two groups of data to be analyzed in ascending or descending order to obtain the sorting position of each datum and then calculate the correlation coefficient between the sorting positions of the two data groups. Spearman’s calculation formula is as follows:

(2) $$\rho =1-\frac{6\sum_{i=1}^{n}d_{i}{}^{2}}{n(n^{2}-1)}$$

where $d_{i}$ is the difference in the sorting positions of the two data groups and *n* is the number of samples.

#### 2.2.2. Feature Vector Length Alignment Method Based on Original Feature Vector Filtering and Fusion

**Figure 2:**
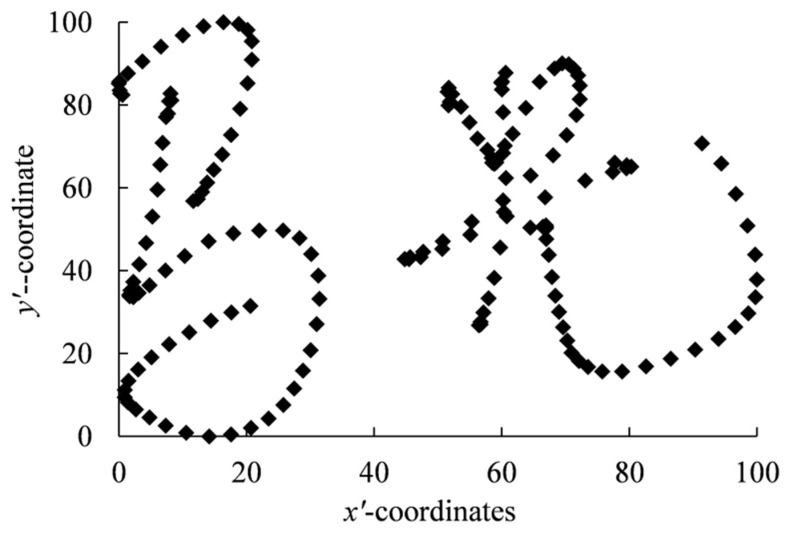
Normalized signature.

Before filtering the original feature vector, in order to eliminate the impact of different signature sizes and positions on feature extraction, the signature was normalized using the linear function normalization Formula (3), which normalizes the *x* coordinate and *y* coordinate of the original signature trajectory to [0, 100]. The normalized signature is shown in Figure 2.

(3) $$x^{\prime }=\frac{x-x_{\min}}{x_{\max}-x_{\min}}\times 100,\;y^{\prime }=\frac{y-y_{\min}}{y_{\max}-y_{\min}}\times 100$$

where x and y are the original coordinates of signature trajectory points; $x_{\max}$ and $x_{\min}$ are the maximum and minimum values of all x in the signature trajectory point; $y_{\max}$ and $y_{\min}$ are the maximum and minimum values of all y in the signature trajectory point; and $x^{\prime }$ and $y^{\prime }$ are normalized coordinates.

Based on signature normalization, we first divided the signature trajectory into five parts according to the intervals of the x coordinates of the signature trajectory—[0, 20), [20, 40), [40, 60), [60, 80), and [80, 100]—then filtered out the original feature data corresponding to each part, and then summed the original feature data of this part as new feature data.

Still taking the SVC 2004 dataset as an example, let the original pen pressure feature vector of 20 feature vector signatures of user1 in the SVC 2004 dataset be $p_{1},\ldots ,\;p_{20}$. Table 1 shows the new pen pressure feature vectors of 20 genuine signatures after length alignment. Let *N* be the total number of samples in the current signature’s original pen pressure feature vector. Then, $p_{11}^{\prime }=\sum_{k=1}^{N}p_{1k},\;x\in \left[0,\;20\right)$ is the sum of the original pen pressure feature data in the first interval [0, 20) filtered from the original pen pressure feature data of the first genuine signature; $p_{12}^{\prime }=\sum_{k=1}^{N}p_{1k},\;x\in \left[20,\;40\right)$ is the sum of the original pen pressure feature data located in the second interval [20, 40) filtered from the original pen pressure feature data of the first genuine signature, and so on, resulting in a new pen pressure feature vector of the first genuine signature as $p_{1}^{\prime }={\left[p_{11}^{\prime },\;p_{12}^{\prime },\;p_{13}^{\prime },\;p_{14}^{\prime },\;p_{15}^{\prime }\right]}^{⊤}$. Correspondingly, the new pen pressure feature vector for 20 genuine signatures can be derived as $p_{1}^{\prime },\ldots ,\;p_{20}^{\prime }$.

**Figure 3:**
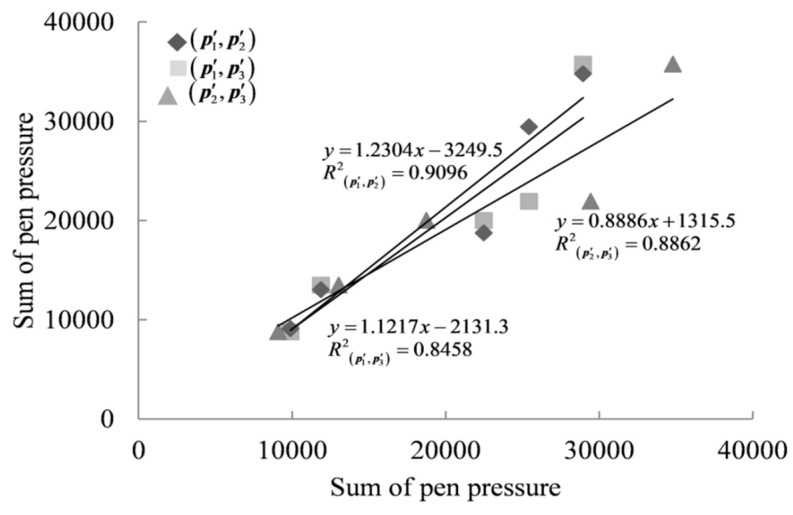
Linear relationship of ($p_{1}^{\prime },\;p_{2}^{\prime }$), ($p_{1}^{\prime },\;p_{3}^{\prime }$), and ($p_{2}^{\prime },\;p_{3}^{\prime }$).

After unifying the length of the feature vector, in order to calculate the correlation coefficient between the two new feature vectors, we first conducted a normal test on the new feature data. Considering that the length of the new feature vector in this paper was five, we selected the Shapiro–Wilk normality test method suitable for a small sample size normal test [48,49], and the results showed that the new feature vector data generally conformed to the normal distribution. After confirming that the feature vectors satisfied a normal distribution, it was necessary to determine further whether they satisfied a linear relationship. In this paper, a total of 553,520 pairs of new feature vectors generated from the xLongSignDB dataset and the SVC 2004 dataset were analyzed by regression analysis. Taking the original pen pressure feature vectors $p_{1}$, $p_{2}$, and $p_{3}$ of USER1′s genuine signature 1, genuine signature 2, and genuine signature 3 in the SVC 2004 dataset as an example, the new pen pressure feature vectors $p_{1}^{\prime }$, $p_{2}^{\prime }$, and $p_{3}^{\prime }$ were analyzed by regression. Taking the new pen pressure feature vectors $p_{1}^{\prime }$ and $p_{2}^{\prime }$ as an example, in order to achieve the regression, it was necessary to use $p_{1}^{\prime }$ as the independent variable and $p_{2}^{\prime }$ as the dependent variable and then use the least squares approximation method to construct the linear prediction function by constantly adjusting the intercept and slope to obtain a higher goodness-of-fit *R*^2^. After the analysis, it was found that the regression equation of ($p_{1}^{\prime }$, $p_{2}^{\prime }$) was y=1.2304x−3249.5, and the goodness-of-fit *R*^2^ was 0.9096, which indicates that ($p_{1}^{\prime }$, $p_{2}^{\prime }$) met a linear relationship. The linear relationship between each new pen pressure feature vector is shown in Figure 3.

After determining that each new characteristic data met the normal distribution and linear relationship, the Pearson correlation analysis method could calculate the Pearson correlation coefficient between the two feature vectors. The calculation formula is as follows:

(4) $$r=\frac{\mathrm{cov}(x_{i},\;y_{j})}{\sigma_{x_{i}}\sigma_{y_{j}}}=\frac{\sum_{i=j=1}^{n}\left(x_{i}-\bar{x})(y_{j}-\bar{y}\right)}{\sqrt{\sum_{i=1}^{n}{\left(x_{i}-\bar{x}\right)}^{2}}\sqrt{\sum_{j=1}^{n}{\left(y_{j}-\bar{y}\right)}^{2}}}$$

where $x_{i}$ and $y_{j}$ are two sets of feature vectors with length *n*, and $\bar{x}$ and $\bar{y}$ are the mean values of samples in feature vectors $x_{i}$ and $y_{j}$, respectively. As shown in Table 1, taking the new pen pressure feature vectors $p_{m}^{\prime }$ and $p_{n}^{\prime }$ of the *m*-th and *n*-th genuine signatures of user1 in the SVC 2004 dataset as an example, after length alignment, Equation (4) becomes as follows:

(5) $$r_{p_{m}^{\prime }}{}_{p_{n}^{\prime }}=\frac{\sum_{i=j=1}^{5}\left(p_{mi}^{\prime }-\bar{p_{m}^{\prime }})(p_{nj}^{\prime }-\bar{p_{n}^{\prime }}\right)}{\sqrt{\sum_{i=1}^{5}{\left(p_{mi}^{\prime }-\bar{p_{m}^{\prime }}\right)}^{2}}\sqrt{\sum_{j=1}^{5}{\left(p_{nj}^{\prime }-\bar{p_{n}^{\prime }}\right)}^{2}}}$$

where $p_{mi}^{\prime }$ represents the sum of the original pen pressure feature data in the *i*-th interval filtered from the original pen pressure feature data of the *m*-th genuine signature, and $p_{nj}^{\prime }$ represents the sum of the original pen pressure feature data in the *j*-th interval filtered from the original pen pressure feature data of the *n*-th genuine signature. $\bar{p_{m}^{\prime }}$ and $\bar{p_{n}^{\prime }}$ are the mean values of the new pen pressure feature data in the two new pen pressure feature vectors $p_{m}^{\prime }$ and $p_{n}^{\prime }$.

### 2.3. Online Signature Verification

Based on the above two feature vector length alignment methods, we combined Formulas (2) and (5) to calculate the correlation coefficients between the new feature vectors in the genuine signature and between the genuine and forged signatures in the xLongSignDB dataset and SVC 2004 dataset, respectively. On this basis, we divided the training and test samples. When dividing the training and test samples, we used the correlation coefficients between the new feature vectors of each writer’s first 10 genuine signatures in the SVC 2004 dataset as the training samples. For the test samples, we used the correlation coefficients between the new feature vectors of the last 10 genuine signatures and the new feature vectors of the first 10 genuine signatures, and the correlation coefficients between the new feature vectors of the 20 forged signatures and the new feature vectors of the first 10 genuine signatures. In the xLongSignDB dataset, the correlation coefficients between the new feature vectors of each writer’s initial 23 authentic signatures were employed as training samples. Additionally, the correlation coefficients between the new feature vectors of the final 23 genuine signatures and the new feature vectors of the first 23 genuine signatures, as well as the correlation coefficients between the new feature vectors of the 10 forged signatures and the new feature vectors of the first 23 genuine signatures, were used as test samples. As an example, the correlation coefficients between the new pen pressure feature vectors of the 40 signatures (20 genuine and 20 forged signatures) of USER1 in the SVC 2004 dataset are shown in Table 2.

After obtaining the correlation coefficients between the new feature vectors, we introduced the Gaussian density function to model these correlation coefficients, as shown in Equation (6).

(6) $$f(x_{i})=\frac{1}{\sqrt{2\pi }\sigma }e^{-\frac{{\left(x_{i}-\mu \right)}^{2}}{2\sigma^{2}}}$$

The $x_{i}$ in Equation (6) is the correlation coefficient between the *i*-th new feature vector, and *μ* and *σ* are the mean and standard deviation of the training samples, respectively. For the final signature verification in this paper, all the calculated correlation coefficients were first substituted into Equation (6) to obtain the function value corresponding to each correlation coefficient. Then, considering that the mean is a widely used statistic, it can serve as an intuitive representation of either the central tendency or the general pattern of sample distribution [54]. Therefore, we used the mean value of all functions corresponding to the training sample as the discrimination threshold for signature verification. Taking Table 2 as an example, the discrimination threshold is $\delta =average\left(f(r_{p_{1}^{\prime }}{}_{p_{1}^{\prime }}),\ldots ,\;f(r_{p_{10}^{\prime }}{}_{p_{10}^{\prime }})\right)$. After obtaining the discrimination threshold, take the average value of the function value of the test sample by column, then $\left\{t_{p_{11}^{\prime }}=average\left(f(r_{p_{11}^{\prime }}{}_{p_{1}^{\prime }}),\ldots ,\;f(r_{p_{11}^{\prime }}{}_{p_{10}^{\prime }})\right),\ldots ,\;t_{p_{40}^{\prime }}=average\left(f(r_{p_{40}^{\prime }}{}_{p_{1}^{\prime }}),\ldots ,\;f(r_{p_{40}^{\prime }}{}_{p_{10}^{\prime }})\right)\right\}$, and then compare ($t_{p_{11}^{\prime }},\ldots ,\;t_{p_{40}^{\prime }}$) with the discrimination threshold δ. If it is greater than δ, it is judged that this signature is genuine; otherwise, it is forged. In this paper, we examined the signature verification performance of each feature using the false rejection rate (*FRR*), the false acceptance rate (*FAR*), and accuracy (*ACC*), where *FRR* is the ratio of the number of genuine signatures that are rejected incorrectly to the total number of genuine signatures; *FAR* is the ratio of the number of forged signatures that are accepted incorrectly to the total number of forged signatures; and *ACC* is the ratio of the number of signatures that are recognized correctly to the total number of signatures.

## 3. Experimental Results and Analysis

According to the above signature verification method, the signature verification performance based on the *X* coordinate feature (*X*), *Y* coordinate feature (*Y*), pen pressure feature (*P*), pen tilt feature (*T*), and pen azimuth feature (*A*) was investigated. The experimental results are shown in Table 3 and Table 4. Table 3 shows the signature verification performance of each feature after filling in the missing value of the original feature vector.

It can be seen from Table 3 that among the three missing value-filling methods, signature verification performance after multiple imputation (MI) was better, with an average accuracy of 83.66% on the xLongSignDB dataset and 88.16% on the SVC 2004 dataset. On the other hand, signature verification performance after filling with zero was poor, with an average accuracy of 73.56% on the xLongSignDB dataset and 72.81% on the SVC 2004 dataset. In addition, among the five features, signature verification performance based on the pen pressure feature was the best, followed by signature verification performance with the pen tilt feature and the pen azimuth feature, while signature verification performance based on the *X* coordinate feature and the *Y* coordinate feature was the worst. It can also be seen from Table 3 that signature verification accuracy with the pen pressure feature after multiple imputation (MI) reached 92.58% on the xLongSignDB dataset and 93.46% on the SVC 2004 dataset. In addition, the pen pressure feature had the highest average accuracy of 86.83% on the xLongSignDB dataset with the three missing value-filling methods (mean values of 87.46%, 92.58%, and 80.46% in Table 3).

Table 4 shows the signature verification performance after filtering and fusion of the original feature vectors. From Table 4 it can be seen that the signature verification performance based on the pen pressure feature was superior on both datasets.

The combined data from Table 3 and Table 4 revealed that both the *X* coordinate and *Y* coordinate features performed poorly in signature verification when using the two feature vector length alignment methods. Specifically, when filled with zero, the *X* coordinate and *Y* coordinate had the lowest average accuracy on the xLongSignDB datasets and the SVC 2004 datasets with rates of 66.22% (mean values of 66.88% and 65.56% in Table 3) and 64.04% (mean values of 64.37% and 63.70% in Table 3), respectively. In addition, signature verification performance with pen tilt and pen azimuth features using two different feature vector length alignment methods is at a medium level. After filtering and fusion, the average accuracy of pen tilt and pen azimuth features was the lowest on the xLongSignDB and SVC 2004 datasets, with results of 73.94% (mean values of 74.39% and 73.48% in Table 4) and 75.14% (mean values of 77.32% and 72.96% in Table 4), respectively. It is worth noting that signature verification performance of the pen pressure feature using the two feature vector length alignment methods was impressive. After multiple imputation (MI), the average accuracy of the pen pressure feature was the highest on the xLongSignDB and SVC 2004 datasets, reaching 93.02% (mean values of 92.58% and 93.46% in Table 3). Conversely, the average accuracy of the pen pressure feature was the lowest on the xLongSignDB and SVC 2004 datasets when filled with zero. However, it still reached 80.94% (mean values of 80.46% and 81.41% in Table 3). Furthermore, comparing the results presented in Table 3 and Table 4 revealed that the average accuracy of signature verification was the lowest for the five features after filling with zero. This method produced an average accuracy of 73.56% on the xLongSignDB dataset and 72.81% on the SVC 2004 dataset. The above two results were close to the average signature verification accuracy of the five features after filtering and fusion on the xLongSignDB datasets (77.03%) and SVC 2004 datasets (73.17%).

Based on the above results, it can be concluded that each feature’s signature verification comprehensive performance was better after mean imputation (MEI) and multiple imputation (MI); conversely, each feature’s signature verification comprehensive performance was worse after filling with zero and filtering and fusion. The explanation for this finding could be that mean imputation (MEI) and multiple imputation (MI) had minimal impact on the original feature vectors during the process of feature vector length alignment, resulting in maximum data preservation in its original form. However, the method of feature vectors with filtering and fusion altered their original form considerably, potentially impacting the calculation of correlation coefficients between features. On the other hand, both mean imputation (MEI) and multiple imputation (MI) methods used the original data to generate the newly filled data. However, the method of filling the missing parts of the data directly with zero did not actually use the original feature data and ignored the original characteristics of the data. Furthermore, signature verification performance improved when using the pen pressure, pen tilt, and pen azimuth features, indicating that increasing the weight of these features in the multi-feature fusion process could further improve signature verification performance.

Considering that some studies use the equal error rate (*EER*) as the primary performance index, in this paper, *EER* was also calculated, and a smaller *EER* represented better overall performance of the algorithm [1]. A comparison of this work with existing methods is shown in Table 5.

Table 5 compares the performance of online signature verification using uni-feature and multi-feature fusion, respectively. For uni-features, the results of the experiments presented in this paper are superior to those found in the existing literature. Furthermore, previous experiments have shown that signature verification performance using pen pressure features is superior, a finding that is consistent with our findings. In contrast to previous multi-feature fusion studies, improved signature verification accuracy using only the pen pressure feature was demonstrated (93.46%). It outperformed both the signature verification accuracy achieved by fusing the *X* and *Y* features (89.30%) and that achieved by fusing all three features (*X* coordinate, *Y* coordinate, and pen pressure features) (80.05%). Moreover, even though the signature verification accuracy of the Y coordinate feature was the lowest in this paper (84.15%), it was also better than the signature verification accuracy after fusing the *X* coordinate, *Y* coordinate, and pen pressure features (80.05%). These results reveal that using a certain uni-feature improved signature verification performance compared to that when fusing certain features. However, it was also found that certain features are unsuitable for use alone, such as the *X* coordinate and *Y* coordinate features.

Furthermore, Table 5 indicates that this paper’s experimental findings are marginally inferior to those of the methods proposed by Cpalka et al. [56] and Chandra et al. [57]. This may indicate that multi-feature fusion can alleviate the problem of limited individual information attached to a uni-feature to a certain extent. However, there are uncertainties and challenges associated with multi-feature fusion. Table 5 shows the results of experiments fusing different numbers and types of features. However, we found it difficult to draw definitive conclusions. For instance, as seen in Table 5, the accuracy declined after fusing three features (80.05%) compared to that when fusing two features (89.30%), and the accuracy also decreased after fusing five features (94%) compared to that when fusing four features (96.52%). Undoubtedly, the verification algorithm used may contribute to this phenomenon, but the quantity and type of the fused features must be considered during the multi-feature fusion process. Furthermore, the fusion of multi-features may be restricted by both the device and the specific scenarios. Accomplishing feature fusion presents a challenge in cases where the device cannot extract multi-features simultaneously. However, while using uni-features may occasionally result in slightly lower signature verification accuracy than after feature fusion, it can also reduce computational costs compared to using multi-features, and uni-features are less likely to be limited by device or scenario constraints.

## 4. Conclusions

An online signature verification method based on the correlation coefficient of uni-features was proposed, considering the problems of unequal feature vector length and low signature verification accuracy when using uni-features for online signature verification. Firstly, two feature vector length alignment methods were proposed, and the correlation analysis method was determined by determining the distribution type of feature data. Then, the correlation coefficients between the same feature vectors were calculated. Finally, a Gaussian density function model was used for signature verification. The experimental results showed that the comprehensive performance of each feature’s signature verification was better after mean imputation (MEI) and multiple imputation (MI). The experimental results also showed that signature verification performance based on the pen pressure feature was the best among that of the five features, with an average accuracy of 86.83% on the xLongSignDB dataset. In the future, we will expand the range of features to be investigated, experiment with additional algorithms, and evaluate their performance in a broader selection of signature datasets to minimize potential constraints due to feature and dataset types and enhance signature verification accuracy.

## Figures and Tables

**Table 1:** New pressure feature vectors of 20 genuine signatures.

New Pen Pressure Feature Vector	[0, 20)	[20, 40)	[40, 60)	[60, 80)	[80, 100]
$p_{1}^{\prime }$	$p_{11}^{\prime }=\sum_{k=1}^{N}p_{1k},\;x\in \left[0,\;20\right)$	$p_{12}^{\prime }=\sum_{k=1}^{N}p_{1k},\;x\in \left[20,\;40\right)$	$p_{13}^{\prime }=\sum_{k=1}^{N}p_{1k},\;x\in \left[40,\;60\right)$	$p_{14}^{\prime }=\sum_{k=1}^{N}p_{1k},\;x\in \left[60,\;80\right)$	$p_{15}^{\prime }=\sum_{k=1}^{N}p_{1k},\;x\in \left[80,\;100\right]$
⋮	⋮	⋮	⋮	⋮	⋮
$p_{m}^{\prime }$	$p_{m1}^{\prime }=\sum_{k=1}^{N}p_{mk},\;x\in \left[0,\;20\right)$	$p_{m2}^{\prime }=\sum_{k=1}^{N}p_{mk},\;x\in \left[20,\;40\right)$	$p_{m3}^{\prime }=\sum_{k=1}^{N}p_{mk},\;x\in \left[40,\;60\right)$	$p_{m4}^{\prime }=\sum_{k=1}^{N}p_{mk},\;x\in \left[60,\;80\right)$	$p_{m5}^{\prime }=\sum_{k=1}^{N}p_{mk},\;x\in \left[80,\;100\right]$
$p_{n}^{\prime }$	$p_{n1}^{\prime }=\sum_{k=1}^{N}p_{nk},\;x\in \left[0,\;20\right)$	$p_{n2}^{\prime }=\sum_{k=1}^{N}p_{nk},\;x\in \left[20,\;40\right)$	$p_{n3}^{\prime }=\sum_{k=1}^{N}p_{nk},\;x\in \left[40,\;60\right)$	$p_{n4}^{\prime }=\sum_{k=1}^{N}p_{nk},\;x\in \left[60,\;80\right)$	$p_{n5}^{\prime }=\sum_{k=1}^{N}p_{nk},\;x\in \left[80,\;100\right]$
⋮	⋮	⋮	⋮	⋮	⋮
$p_{20}^{\prime }$	$p_{20\;1}^{\prime }=\sum_{k=1}^{N}p_{20k},\;x\in \left[0,\;20\right)$	$p_{20\;2}^{\prime }=\sum_{k=1}^{N}p_{20k},\;x\in \left[20,\;40\right)$	$p_{20\;3}^{\prime }=\sum_{k=1}^{N}p_{20k},\;x\in \left[40,\;60\right)$	$p_{20\;4}^{\prime }=\sum_{k=1}^{N}p_{20k},\;x\in \left[60,\;80\right)$	$p_{20\;5}^{\prime }=\sum_{k=1}^{N}p_{20k},\;x\in \left[80,\;100\right]$

Bold represents the feature vectors.

**Table 2:** Correlation coefficient between new pressure feature vectors on SVC 2004 signature dataset.

New Pen Pressure Feature Vector	$p_{1}^{\prime }$	…	$p_{10}^{\prime }$	$p_{11}^{\prime }$	…	$p_{20}^{\prime }$	$p_{21}^{\prime }$	…	$p_{40}^{\prime }$
$p_{1}^{\prime }$	$r_{p_{1}^{\prime }}{}_{p_{1}^{\prime }}$	…	$r_{p_{10}^{\prime }}{}_{p_{1}^{\prime }}$	$r_{p_{11}^{\prime }}{}_{p_{1}^{\prime }}$	…	$r_{p_{20}^{\prime }}{}_{p_{1}^{\prime }}$	$r_{p_{21}^{\prime }}{}_{p_{1}^{\prime }}$	…	$r_{p_{40}^{\prime }}{}_{p_{1}^{\prime }}$
$p_{2}^{\prime }$	$r_{p_{1}^{\prime }}{}_{p_{2}^{\prime }}$	…	$r_{p_{10}^{\prime }}{}_{p_{2}^{\prime }}$	$r_{p_{11}^{\prime }}{}_{p_{2}^{\prime }}$	…	$r_{p_{20}^{\prime }}{}_{p_{2}^{\prime }}$	$r_{p_{21}^{\prime }}{}_{p_{2}^{\prime }}$	…	$r_{p_{40}^{\prime }}{}_{p_{2}^{\prime }}$
⋮	⋮	⋱	⋮	⋮	⋱	⋮	⋮	⋱	⋮
$p_{10}^{\prime }$	$r_{p_{1}^{\prime }}{}_{p_{10}^{\prime }}$	…	$r_{p_{10}^{\prime }}{}_{p_{10}^{\prime }}$	$r_{p_{11}^{\prime }}{}_{p_{10}^{\prime }}$	…	$r_{p_{20}^{\prime }}{}_{p_{10}^{\prime }}$	$r_{p_{21}^{\prime }}{}_{p_{10}^{\prime }}$	…	$r_{p_{40}^{\prime }}{}_{p_{10}^{\prime }}$

**Table 3:** Signature verification performance of each feature based on original feature vectors filled with missing values.

Filling Method	Feature	xLongSignDB DataSet			SVC 2004 DataSet		
		*FRR*	*FAR*	*ACC*	*FRR*	*FAR*	*ACC*
MEI	*X*	0.297	0.183	73.78%	0.287	0.278	71.93%
	*Y*	0.309	0.272	70.22%	0.317	0.292	69.93%
	*P*	0.115	0.148	87.46%	0.200	0.134	84.07%
	*T*	0.142	0.186	84.43%	0.207	0.219	78.52%
	*A*	0.132	0.176	85.48%	0.238	0.201	78.67%
MI	*X*	0.287	0.176	74.71%	0.282	0.061	86.51%
	*Y*	0.215	0.257	77.32%	0.269	0.103	84.15%
	*P*	0.058	0.110	92.58%	0.082	0.057	93.46%
	*T*	0.133	0.162	85.79%	0.240	0.073	87.11%
	*A*	0.114	0.138	87.88%	0.207	0.053	89.56%
Zero	*X*	0.385	0.207	66.88%	0.322	0.356	65.56%
	*Y*	0.376	0.310	64.37%	0.333	0.378	63.70%
	*P*	0.147	0.307	80.46%	0.273	0.143	81.41%
	*T*	0.166	0.342	78.58%	0.211	0.248	76.44%
	*A*	0.179	0.328	77.53%	0.249	0.221	76.96%

**Table 4:** Signature verification performance of each feature based on original feature vectors with filtering and fusion.

Feature	xLongSignDB DataSet			SVC 2004 DataSet		
	*FRR*	*FAR*	*ACC*	*FRR*	*FAR*	*ACC*
*X*	0.279	0.221	73.87%	0.400	0.222	71.85%
*Y*	0.297	0.252	71.68%	0.415	0.260	68.81%
*P*	0.142	0.072	87.88%	0.298	0.170	78.74%
*T*	0.285	0.189	74.39%	0.302	0.247	73.48%
*A*	0.654	0.203	77.32%	0.278	0.267	72.96%

**Table 5:** Comparison of signature verification methods.

Literature	DataSet	Method	Feature	*FRR*	*FAR*	*EER*	*ACC*
Lei et al. [21]	SVC	DTW	*X*	-	-	0.251	-
			*Y*	-	-	0.187	-
			*P*	-	-	0.256	-
			*T*	-	-	0.291	-
			*A*	-	-	0.266	-
Friedman et al. [22]	SVC	DTW + Feature Normalization	*P*	-	-	-	75.40%
			*T*	-	-	-	57.50%
			*A*	-	-	-	73.30%
Saleem et al. [55]	SVC	Signer-Dependent Sampling Frequency	*X*	-	-	0.178	-
			*Y*	-	-	0.163	-
			*P*	-	-	0.138	-
Cpałka et al. [56]	SVC	Signature Partitioning + Fuzzy Classifier	(*X*,*Y*)	0.109	0.105	-	89.30%
Shen et al. [6]	SVC	Siamese Neural Network	(*X*,*Y*,*P*)	0.186	0.208	-	80.05%
Chandra et al. [57]	SVC	Random Forest	(*X*,*Y*,*P*,*T*,*A*)	0.063	0.058	-	94.00%
Zalasiński et al. [58]	xLongSignDB	Signature Partitioning + Fuzzy Classifier	(*X*,*Y*,*P*,*V*)	0.029	0.041	-	96.52%
Our paper	SVC	Multiple Imputation + Correlation Coefficient + Gaussian Density Function	*X*	0.282	0.061	0.139	86.51%
			*Y*	0.269	0.103	0.187	84.15%
			*P*	0.082	0.057	0.076	93.46%
			*T*	0.240	0.073	0.138	87.11%
			*A*	0.207	0.053	0.109	89.56%
Our paper	xLongSignDB	Multiple Imputation + Correlation Coefficient + Gaussian Density Function	*X*	0.287	0.176	0.270	74.71%
			*Y*	0.215	0.257	0.262	77.32%
			*P*	0.058	0.110	0.089	92.58%
			*T*	0.133	0.162	0.172	85.79%
			*A*	0.114	0.138	0.148	87.88%

## Data Availability

Data are contained within the article.
